# The Long Non-Coding RNA *MIR503HG* Enhances Proliferation of Human ALK-Negative Anaplastic Large-Cell Lymphoma

**DOI:** 10.3390/ijms19051463

**Published:** 2018-05-14

**Authors:** Po-Shuan Huang, I-Hsiao Chung, Yang-Hsiang Lin, Tzu-Kang Lin, Wei-Jan Chen, Kwang-Huei Lin

**Affiliations:** 1 Department of Biochemistry, College of Medicine, Chang Gung University, Taoyuan 333, Taiwan; leo_6813@msn.com (P.-S.H.); isiou2007game@gmail.com (I.-H.C.); yhlin0621@cgmh.org.tw (Y.-H.L.); 2 Liver Research Center, Chang Gung Memorial Hospital, Linkou, Taoyuan 333, Taiwan; 3 Neurosurgery, Fu Jen Catholic University Hospital and School of Medicine, Fu Jen Catholic University, New Taipei City 24250, Taiwan; tklin100@gmail.com; 4 Cardiovascular Division, Chang Gung Memorial Hospital, Chang Gung University College of Medicine, Taoyuan 333, Taiwan; wjchen@adm.cgmh.org.tw; 5 Research Center for Chinese Herbal Medicine, College of Human Ecology, Chang Gung University of Science and Technology, Taoyuan 333, Taiwan

**Keywords:** ALK-negative ALCL, *MIR503HG*, proliferation

## Abstract

Anaplastic lymphoma kinase (ALK)-negative anaplastic large-cell lymphoma (ALCL) is a rare type of highly malignant, non-Hodgkin lymphoma. Currently, only a few gene rearrangements have been linked to ALK-negative ALCL progression. However, the specific molecular mechanisms underlying the growth of ALK-negative ALCL tumors remain unclear. Here, we investigated aberrantly expressed, long non-coding RNAs (lncRNAs) in ALK-negative ALCL and assessed their potential biological function. *MIR503HG* (*miR-503* host gene) was highly expressed in ALK-negative cell lines and was significantly upregulated in tumors in mice formed from ALK-negative ALCL cell lines. Depletion of *MIR503HG* suppressed tumor cell proliferation in vivo and in vitro; conversely, its overexpression enhanced tumor cell growth. *MIR503HG*-induced proliferation was mediated by the induction of *microRNA-503* (*miR*-*503*) and suppression of Smurf2, resulting in stabilization of the tumor growth factor-β receptor (TGFBR) and enhanced tumor cell growth. Collectively, these findings support a potential role for *MIR503HG* in cancer cell proliferation through the *miR-503*/Smurf2/TGFBR axis and indicate that *MIR503HG* is a potential marker in ALK-negative ALCL.

## 1. Introduction

Anaplastic lymphoma kinase (ALK)-negative anaplastic large cell lymphoma (ALCL) is a highly malignant non-Hodgkin lymphoma of CD30-positive T-cells that lacks chromosomal rearrangements of the *ALK* gene encoding a receptor tyrosine kinase [1,2,3]. The Nucleophosmin (NPM)-ALK fusion protein resulting from translocation involving the *ALK* gene on 2p23, as well as the oncogenic role of these fusion proteins, has been studied extensively in ALK-positive ALCL [4,5,6]. In contrast, the pathogenic mechanism of ALK-negative ALCL is poorly understood. Moreover, it is difficult to distinguish ALK-negative ALCL from other CD30-positive peripheral T-cell lymphomas (PTCLs) because genetic biomarkers are lacking. Ferreri et al. reported that survival rates are higher in patients with ALK-positive ALCL than in ALK-negative patients [7]. Recent studies have also shown that ALK-negative ALCL is a genetically heterogeneous disease whose outcome varies widely depending on genetic subtype [8]. Therefore, identifying an effective marker is necessary for early diagnosis and treatment of ALK-negative ALCL.

Long non-coding RNAs (lncRNAs) are transcripts longer than 200 nucleotides in length that do not encode a protein. Several lncRNAs that are aberrantly expressed in tumors have been reported to play an important role in cancer [9,10,11]. Clinically, lncRNAs associated with survival or prognosis are potential therapeutic targets [12]. However, the clinical significance of lncRNA expression in ALK-negative ALCL development is largely unclear. A previous study identified aberrantly expressed lncRNAs in ALCL specimens using oligonucleotide arrays. We have investigated these overexpressed lncRNAs for their potential function in ALCL progression, focusing on the top five non-coding genes (fold change > 3.5): BMS1P20, LINC01013, *MIR503HG*, RNF144A-AS1 and CACNA1G-AS1 [13].

Transforming growth factor β (TGF-β) signaling pathways are important in the regulation of cellular proliferation, migration, angiogenesis, and survival [14]. TGF-β ligands have been used as diagnostic, prognostic or predictive markers for many different cancers, including lymphoma [15]. TGF-β signaling is mediated by its cognate receptor, transforming growth factor beta receptor (TGFBR), a transmembrane serine/threonine kinase. Interaction of TGF-β with the receptor leads to phosphorylation and activation of TGFBR, resulting in stimulation of the SMADs signal pathway [16].

In this study, we investigated the role of lncRNAs in regulating proliferative activity and explored underlying molecular mechanisms in ALK-negative ALCL. We found that the potential lncRNA *MIR503HG*, a host gene of the microRNA miRNA-503, was highly expressed in tumors induced in mice by ALK-negative ALCL cells compared with those formed from ALK-positive ALCL cells. Accordingly, we hypothesized that the functions of *MIR503HG* are associated with cancer progression. Specifically, our in vitro and in vivo analyses revealed that *MIR503HG*, which has not been linked to human ALK-negative ALCL, is overexpressed in ALK-negative ALCL cell lines and is significantly upregulated in tumor specimens from mice harboring ALK-negative ALCL tumors.

## 2. Results

### 2.1. High Expression of MIR503HG in ALK-Negative ALCL Is Associated with Tumor Proliferation

**Figure 1:**
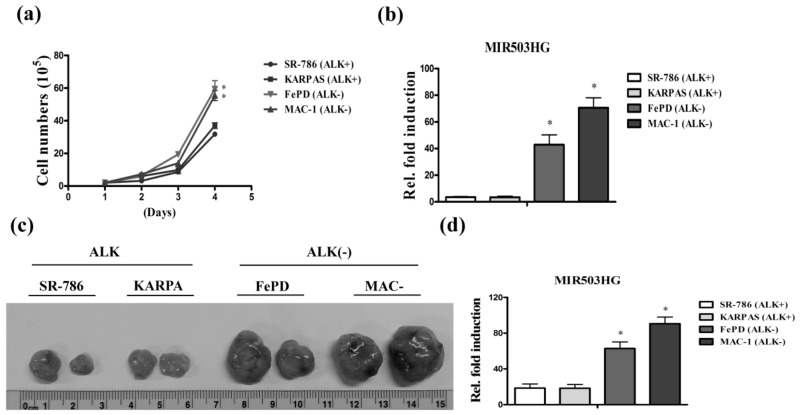
Identification of *MIR503HG* in anaplastic lymphoma kinase (ALK)-negative anaplastic large-cell lymphoma (ALCL). (**a**) The cell growth ability of ALK-positive (SR-786 or KARPAS) and ALK-negative (FePD or MAC-1) cell lines was tested. (**b**) Expression levels of *MIR503HG* were measured by q-RT-PCR. (**c**) Tumors from nude mice subcutaneously injected with ALK-positive or -negative cells were photographed. (**d**) *MIR503HG* expression levels, determined by qRT-PCR. Differences were analyzed using a Kruskal–Wallis test (* *p* < 0.05). Data are presented as means ± SEMs of at least three experiments.

To identify aberrantly expressed lncRNAs and establish their functional correlation with ALK-negative (FePD, MAC-1) and ALK-positive (SR-786, KARPAS) ALCL cell lines, we performed proliferation assays. Notably, we found that *MIR503HG* was highly expressed in faster-growing ALK-negative cell lines compared with ALK-positive cell lines (Figure 1a,b). Tumors from nude mice subcutaneously injected with ALK-negative cells were increased in size (Figure 1c) and showed higher expression levels of *MIR503HG* (Figure 1d) compared with ALK-positive cells. Collectively, these results confirm that *MIR503HG* is highly expressed in ALK-negative ALCL, suggesting a possible role for *MIR503HG* in ALK-negative ALCL cell growth.

### 2.2. MIR503HG Depletion Suppresses ALK-Negative ALCL Proliferation In Vitro and In Vivo

**Figure 2:**
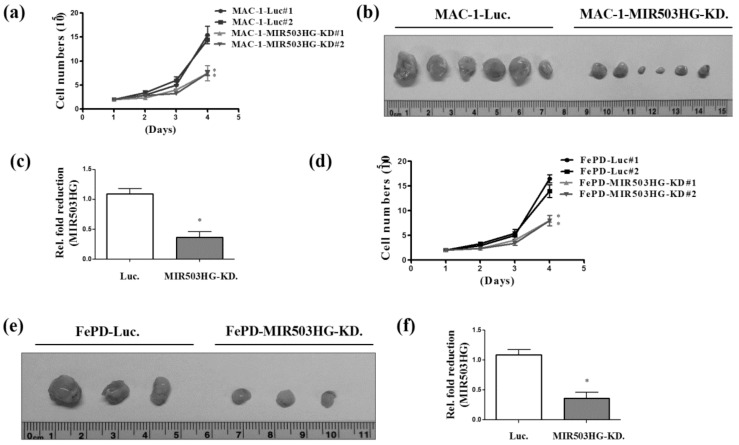
*MIR503HG* depletion suppresses ALK-negative ALCL growth. (**a**) Proliferation ability was analyzed in *MIR503HG*-depleted (*MIR503HG*-KD#1 or KD#2) and control (Luc#1 or Luc#2) MAC-1 cells. (**b**) Tumors from nude mice subcutaneously injected with *MIR503HG*-depleted or control MAC-1 cells were photographed. (**c**) *MIR503HG* expression levels, determined by qRT-PCR. (**d**) Proliferation of FePD cells, assayed under conditions similar to those for MAC-1 cells. (**e**) Tumors from nude mice injected with *MIR503HG*-depleted or control FePD cells were photographed. (**f**) *MIR503HG* expression levels, determined by qRT-PCR. Differences were analyzed using a Kruskal–Wallis test (* *p* < 0.05). Data are presented as means ± SEMs of at least three experiments.

To determine whether *MIR503HG* is capable of affecting cell proliferation in ALK-negative ALCL cells, we first established *MIR503HG*-knockdown and control MAC-1 cells. We found that proliferation was significantly decreased in *MIR503HG*-depleted MAC-1 cells (Figure 2a). Moreover, tumors formed in vivo from *MIR503HG*-depleted MAC-1 cells subcutaneously injected in nude mice were decreased in size (Figure 2b) and showed lower expression levels of *MIR503HG* compared with controls (Figure 2c). These features were recapitulated in *MIR503HG*-depleted FePD cells (Figure 2d) and tumor-bearing nude mice (Figure 2e,f).

### 2.3. MIR503HG Depletion Acts through Suppression of miR-503 to Promote Degradation of the TGF-β Receptor In Vitro and In Vivo

**Figure 3:**
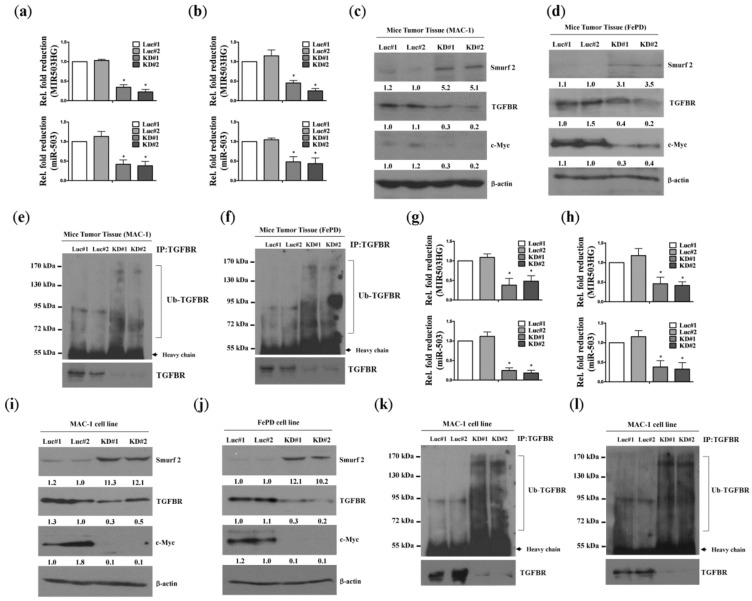
*MIR503HG* depletion promotes degradation of TGFBR. (**a**,**b**) *MIR503HG* and *miR-503* expression levels were determined by qRT-PCR in specimens of tumors from nude mice injected with *MIR503HG*-depleted (KD#1 or KD#2) or control (Luc#1 or Luc#2) MAC-1 (**a**) or FePD (**b**) cells. (**c**,**d**) Smurf2, TGFBR, and c-Myc expression levels were determined. (**e**,**f**) Immunoprecipitation assays of specimens of tumors from nude mice injected with *MIR503HG*-depleted or control MAC-1 (**e**) and FePD (**f**) cells; ubiquitinated TGFBR was analyzed by Western blotting. (**g**,**h**) *MIR503HG* and *miR-503* expression levels in MAC-1 (**g**) and FePD (**h**) cell lines were determined by q-RT-PCR under similar conditions. (**i**,**j**) Smurf2, TGFBR, and c-Myc expression levels in these cell lines were determined. (**k**,**l**) Immunoprecipitation assays of *MIR503HG*-depleted and control MAC-1 (**k**) and FePD (**l**) cells; ubiquitinated TGFBR was analyzed by Western blotting. Differences were analyzed using a Kruskal-Wallis test (* *p* < 0.05). Data are presented as means ± SEMs of at least three experiments.

To further identify the potential mechanism underlying *MIR503HG* regulation of ALK-negative cell proliferation, we analyzed tumors induced in nude mice by the injection of *MIR503HG*-depleted MAC-1 or FePD cells. Cao et al. previously reported that the E3 ubiquitin-protein ligase Smurf2 is a target of *miR-503* [17]. Notably, tumors formed from *MIR503HG*-depleted MAC-1 or FePD cells exhibited a significant decrease in the levels of *miR-503* (Figure 3a,b) as well as TGFBR and c-Myc (a downstream target of TGFBR signaling) compared with controls; effects that were accompanied by an increase in Smurf2 expression (Figure 3c,d) and ubiquitination-dependent, proteasome-mediated TGFBR degradation (Figure 3e,f). Similar results were obtained for *MIR503HG*-depleted MAC-1 and FePD cells in vitro (Figure 3g–l). Together, these data indicate that induction of *miR-503* by *MIR503HG* may serve to protect TGFBR from Smurf2-mediated ubiquitination and subsequent degradation.

### 2.4. MIR503HG Overexpression Promotes ALK-Positive ALCL Growth In Vitro and In Vivo

**Figure 4:**
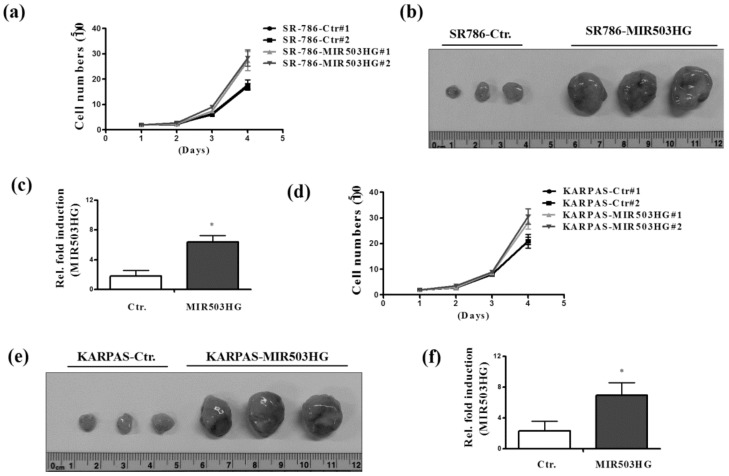
*MIR503HG* overexpression promotes growth of ALK-positive ALCL cells. (**a**) Proliferation ability was analyzed in *MIR503HG*-overexpressing (*MIR503HG*#1 or *MIR503HG*#2) and control (Ctr#1 or Ctr#2) SR-786 cells. (**b**) Tumors from nude mice subcutaneously injected with *MIR503HG*-overexpressing or control SR-786 cells were photographed. (**c**) *MIR503HG* expression levels, determined by qRT-PCR (**d**) Proliferation assays of KARPAS cells performed under similar conditions. (**e**) Tumors from nude mice injected with *MIR503HG*-overexpressing or control KARPAS cells were photographed. (**f**) *MIR503HG* expression levels, determined by qRT-PCR. Differences were analyzed using a Kruskal–Wallis test (* *p* < 0.05). Data are presented as means ± SEMs of at least three experiments.

To further assess the effect of *MIR503HG* on ALK-positive ALCL cell proliferation, we established *MIR503HG*-overexpressing SR-786 and control cell lines. Notably, *MIR503HG*-overexpressing SR-786 cells displayed significantly increased proliferation compared with control cells (Figure 4a). Similarly, nude mice subcutaneously injected with *MIR503HG*-overexpressing SR-786 cells showed increased tumor size (Figure 4b), as well as higher expression levels of *MIR503HG* (Figure 4c) compared with those injected with control cells. Similar results were observed in *MIR503HG*-overexpressing KARPAS cells (Figure 4d) and corresponding tumor-bearing nude mice (Figure 4e,f).

### 2.5. MIR503HG Overexpression Acts through Induction of miR-503 to Stabilize TGFBR and Enhance Cell Proliferation In Vitro and In Vivo

**Figure 5:**
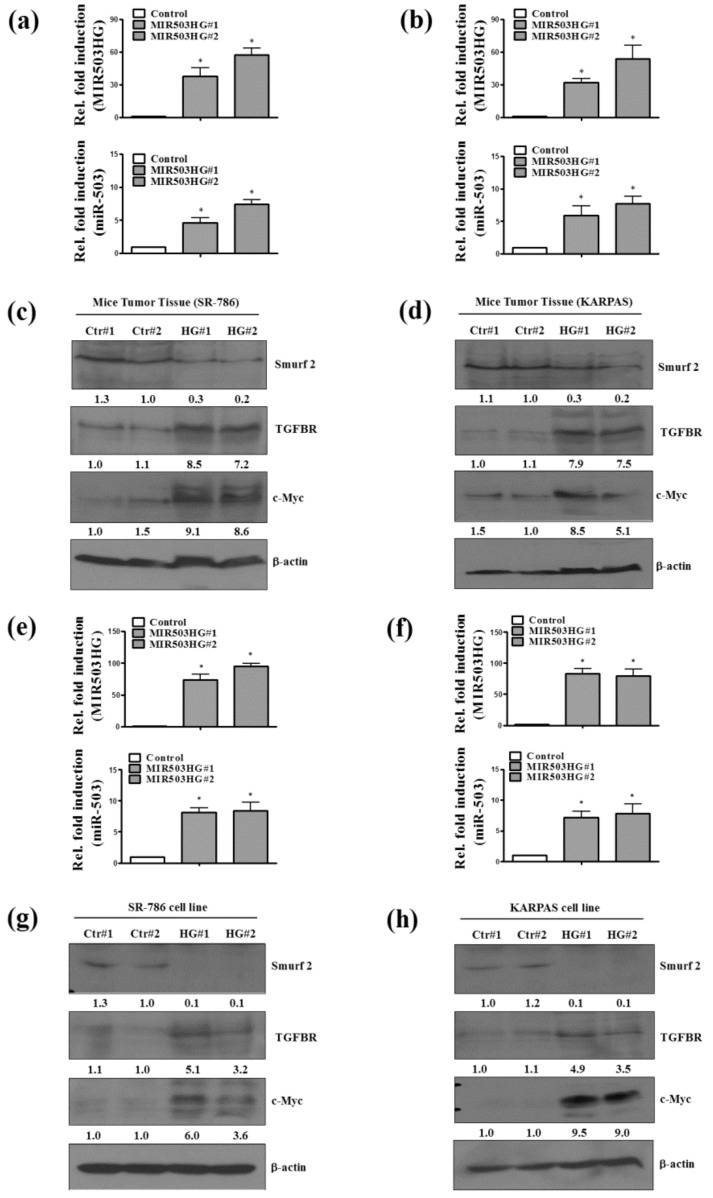
*MIR503HG* overexpression acts through induction of *miR-503* to stabilize TGFBR and enhance cell proliferation. (**a**,**b**) *MIR503HG* and *miR-503* expression levels in specimens of tumors from nude mice injected with *MIR503HG*-overexpressing (*MIR503HG*#1 or *MIR503HG*#2) or control SR-786 and KARPAS cells were determined by qRT-PCR. (**c**,**d**) Smurf2, TGFBR, and c-Myc expression levels were determined by Western blotting. (**e**,**f**) *MIR503HG* and *miR-503* expression levels were determined by qRT-PCR in SR-786 and KARPAS cell lines under similar conditions. (**g**,**h**) Smurf2, TGFBR, and c-Myc expression levels in these cell lines were determined by Western blotting. Differences were analyzed using a Kruskal–Wallis test (* *p* < 0.05). Data are presented as means ± SEMs of at least three experiments.

**Figure 6:**
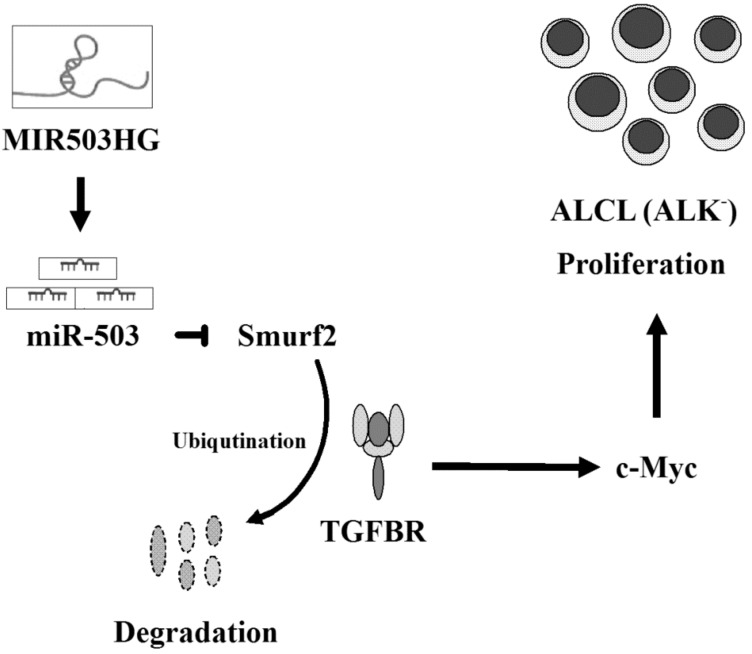
*MIR503HG* promotes ALK-negative ALCL proliferation. Schematic depiction of *MIR503HG* promotion of ALK-negative ALCL proliferation through activation of the *miR-503*/Smurf2/TGFBR cascade. LncRNA *MIR503HG* is highly expressed in ALK-negative cells compared with ALK-positive ALCL cells. Concomitantly, *miR-503* (its host gene *MIR503HG*) is stimulated, decreasing its target Smurf2 (an E3 ubiquitin ligase) and consequently stabilizing TGFBR, thereby inducing c-Myc expression and subsequent cell proliferation. (→: induces)

To further confirm the specific mechanism by which *MIR503HG* regulates ALK-positive cell proliferation, we analyzed tumor specimens from nude mice injected with *MIR503HG*-overexpressing SR-786 or KARPAS cells. Notably, tumors formed from *MIR503HG*-overexpressing cells exhibited a significant increase in *miR-503* (Figure 5a,b), TGFBR, and c-Myc (Figure 5c,d) expression levels. However, Smurf2 expression was decreased (Figure 5c,d) compared with that in control groups. Similar results were obtained with *MIR503HG*-overexpressing SR-786 and KARPAS cells in vitro (Figure 5e–h). Together, these findings indicate that *MIR503HG* may protect TGFBR from Smurf2-mediated ubiquitination and subsequent proteasome-dependent degradation. Collectively, these results support the conclusion that *MIR503HG* promotes lymphoma cell proliferation in ALK-negative ALCL through the *miR-503*/Smurf2/TGFBR pathway (Figure 6).

## 3. Discussion

Our data indicate that *MIR503HG* plays an oncogenic role in ALK-negative ALCL by inducing *miR-503* expression; this increase in *miR-503* leads to Smurf2 degradation and TGFBR stabilization and promotes cell proliferation. The specific pathways involved in ALK-negative ALCL proliferation and the pivotal role of aberrantly expressed *MIR503HG* in ALK-negative ALCL was validated using an in vivo mouse model. We further found that high expression of *MIR503HG* in ALK-negative ALCL specimens is associated with faster cell growth. Both gain- and loss-of-function strategies clearly demonstrated that *MIR503HG* induces proliferation of ALK-negative ALCL cells in vitro and in vivo.

A number of reports have indicated that lncRNAs regulate gene expression through multiple mechanisms at both transcriptional and posttranscriptional levels, including through RNA splicing and epigenetic regulation [18]. Notably, it has been reported that more than 18% of non-protein–coding genes are associated with cancer through unknown functions/mechanisms [19], highlighting the importance of understanding the role of lncRNAs in different cancers. Many abnormally expressed lncRNAs have been reported as potential biomarkers or targets of multiple cancer therapies [20]. In this context, our findings suggest that *MIR503HG* can be used as a novel oncogenic/proliferation marker for ALK-negative ALCL.

Recent studies of ALK-positive ALCL development have focused on NPM1-ALK translocation and pathogenesis [21,22]. Such ALK fusion proteins exhibit aberrant tyrosine kinase activity that enhances cell proliferation and survival [23]. In contrast, the mechanism underlying progression in ALK-negative ALCL, which lacks the NPM1-ALK fusion protein, has remained largely unknown. Several studies have demonstrated the presence of chromosomal rearrangements of *DUSP22* or *TP63*, overexpression of ERBB4, and mutation of JAK1/STAT3 in ALK-negative ALCL [24].

*MIR503HG* is a lncRNA that maps to chromosome X (Xq26), a region enriched for genes associated with human reproduction. A recent study reported that *MIR503HG* is highly expressed in the placenta and other reproductive tissues and may play a role in human reproduction and tumorigenesis [25,26]. Expression levels of *miR*-*503*, an miRNA that neighbors *MIR503HG* on the chromosome, are increased in several cancer cell types and are positively correlated with those of *MIR503HG* [25,27,28,29]. In the current study, we found that *MIR503HG*-induced proliferation was mediated by activation of *miR-503*, but the detailed mechanism is still unclear. To our knowledge, no studies to date have reported an association between *MIR503HG* and ALK-negative ALCL progression and involvement of a *MIR503HG*-mediated pathway in ALK-negative ALCL proliferation.

Smurf2 is an E3 ubiquitin ligase that regulates TGF-β signaling [30]. Previous studies have shown that Smurf2 suppresses the TGFBR via ubiquitin-mediated degradation in cancer cells [31]. Moreover, Smurf2 is directly suppressed by *miR-503*, thereby promoting tumor progression [32]. However, no reports have linked the regulation of lncRNA with the *miR-503*/Smuf2/ TGFBR pathway in ALK-negative ALCL. The current study showed that *MIR503HG* enhances the growth ability of ALK-negative ALCL by protecting and activating components of the *miR-503*/Smuf2/ TGFBR pathway. Collectively, our findings provide novel insight into the role of *MIR503HG* in lymphoma progression and identify a new potential therapeutic target in ALK-negative ALCL.

## 4. Materials and Methods

### 4.1. Mouse Xenograft Model

ALCL cells (1 × 10^6^ cells/200 μL) were injected subcutaneously into the flanks of immunodeficient nude mice. After 2 months, tumor tissues were collected for RNA extraction. At least 2–6 mice per group were used in all animal experiments. Animal experimental protocols (IACUC Approval No: CGU13-105; Period of Protocol: valid from 08/01/2014–07/31/2017) were reviewed and approved by the National Laboratory Animal Center (NLAC). All experiments were performed in accordance with the relevant guidelines and regulations of the Laboratory Animal Center of Chang Gung University, and all efforts were made to minimize animal pain.

### 4.2. Quantitative Reverse Transcription-Polymerase Chain Reaction

Total RNA was collected using the TRIzol reagent (Life Technologies Inc., Carlsbad, CA, USA) and reverse transcribed into cDNA using a Superscript II kit (Life Technologies, Karlsruhe, Germany). *MIR503HG* expression levels were measured by quantitative reverse transcription-polymerase chain reaction (qRT-PCR) using a Real-Time ABI PRISM 7500 system (Applied Biosystems, Foster City, CA, USA). *MIR503HG* was detected using the forward primer *MIR503HG*-F (5′-AAGGAATCCTCTCCCACCATTT-3′) and reverse primer *MIR503HG*-R (5′-ACT CAT TTG GCG GGA AAA C-3′). A single unique sequence corresponding to *MIR503HG* (NCBI Reference Sequence: NR_024607.1 [33]) was detected. *MIR503HG* levels were normalized to those of 18S ribosomal RNA (18S rRNA). *miR-503*-5p was detected using miRNA-specific stem-loop reverse transcription primer (*miR-503*-5p: 5′-CTCAACTGGTGTCGTGGAGTCGGCAATTCAGTTGAGCTG CAGAA-3′) in miRNA real-time reactions. *miR-503*-5p expression was subsequently detected using the specific primer *miR-503*-5p-F (5′-CGGCGGTAGCAGCGGGAACAGT-3′) and universal reverse primer Universal-R (5′-CTGGTGTCGTGGAGTCGGCAATTC-3′). Values were normalized to those of RNU6B (U6 snRNA).

### 4.3. Cell Culture

Human ALK-positive (SR-786, KARPAS) and negative (MAC-1, FePD) ALCL cell lines were cultured in Dulbecco’s modified Eagle’s medium (DMEM) containing 10% fetal bovine serum (FBS) at 37 °C in a humidified 5% CO_2_ incubator.

### 4.4. Immunoblotting

Protein concentrations were determined using a Bradford-based method (Pierce Biotechnology, Rockford, IL, USA). Equal amounts of protein (80 μg/lane) were separated by sodium dodecyl sulfate-polyacrylamide gel electrophoresis (SDS-PAGE) on 8–10% gels (Tris Buffer 1.5 M pH 8.8; Biotools Co., Ltd., Taipei, Taiwan). Separated proteins were transferred to a nitrocellulose membrane (pH 7.9; Amersham Biosciences Inc., Piscataway, NJ, USA) and incubated with different primary antibodies, including anti-Smurf2 (ab53316, Abcam, Cambridge, UK), anti- TGFBR (ab124894, Abcam, Cambridge, UK) and anti-c-Myc (ab32072, Abcam, Cambridge, UK).

### 4.5. Establishment of Stable MIR503HG-Knockdown Cell Lines

*MIR503HG* short hairpin RNAs (shRNAs) were purchased from the National RNAi Core Facility (Institute of Molecular Biology, Academia Sinica, Taipei, Taiwan). Stable *MIR503HG*-knockdown MAC-1 and FePD cell lines infected with lentivirus expressing shRNA against *MIR503HG* were established by culturing cells in selection medium consisting of DMEM/10% FBS containing puromycin (0.3 μg/mL).

### 4.6. Establishment of Stable MIR503HG-Overexpressing Cell Lines

An *MIR503HG* expression plasmid was obtained by PCR-amplifying *MIR503HG* using the specific primers 5′-CGCTGGTACCGAAGGTAGAAGGTGGGGTC-3′ (forward) and 5′-CGCGCTCGAGTAACGGAAATCAAAAGCAGC-3′ (reverse) and cloning the resulting PCR product into the pcDNA3 vector. *MIR503HG*-overexpressing SR-786 and KARPAS cell lines were established by first transfecting cells with the resulting *MIR503HG* expression plasmid using the Lipofectamine reagent (Invitrogen, Waltham, MA, USA). After 24 h, stably expressing cells were selected by culturing transfected cells in the presence of G418 (800 μg/mL). Expression levels of *MIR503HG* RNA were determined by qRT-PCR.

### 4.7. Proliferation Assay

Cell proliferation was evaluated by seeding cells at 5 × 10^4^ cells and then counting cell numbers after several days using a hemocytometer.

### 4.8. Ubiquitination Assay

Whole-cell lysates were extracted after treating cells for 4 h with the proteasome inhibitor MG132 (10 mM). Non-specific binding was eliminated by first incubating cell lysates with protein A/G (Santa Cruz Biotechnology, Santa Cruz, CA, USA) for 1 h, after which lysates were incubated with anti- TGFBR antibody (Abcam, Biotech, Life Science; ab124894) overnight at 4 °C. The next day, lysates were incubated with protein A/G for 1 h at 4 °C. Ubiquitinated TGFBR signals were detected with an anti-ubiquitin antibody (Epitomics Inc., Burlingame, CA, USA).

### 4.9. Statistical Analysis

Data are presented as means ± SEMs of at least three experiments. Statistical analyses were performed using Student’s *t* test or one-way analysis of variance (one-way ANOVA). Where suitable, a Mann–Whitney U or Fisher’s exact test was used; a Kruskal–Wallis test or Pearson’s χ^2^ test was used for comparisons of more than two groups. Spearman’s rank correlation coefficient was used to assess correlations of two different examinations.
